# Automated measurement of inter-arytenoid distance on 4D laryngeal CT: A validation study

**DOI:** 10.1371/journal.pone.0279927

**Published:** 2023-01-18

**Authors:** Andrew Ma, Nandakishor Desai, Kenneth K. Lau, Marimuthu Palaniswami, Terence J. O’Brien, Paari Palaniswami, Dominic Thyagarajan

**Affiliations:** 1 Department of Neuroscience, Central Clinical School, Monash University, Melbourne, Victoria, Australia; 2 Department of Neurology, Alfred Health, Melbourne, Victoria, Australia; 3 Department of Neurology, Monash Health, Melbourne, Victoria, Australia; 4 Department of Electrical and Electronic Engineering, The University of Melbourne, Melbourne, Victoria, Australia; 5 School of Clinical Sciences, Faculty of Medicine, Nursing and Health Sciences, Monash University, Melbourne, Victoria, Australia; 6 Monash Health Imaging, Monash Health, Melbourne, Victoria, Australia; 7 School of Computing and Information Systems, Faculty of Engineering and Information Technology, The University of Melbourne, Melbourne, Victoria, Australia; Museo Storico della Fisica e Centro Studi e Ricerche Enrico Fermi, ITALY

## Abstract

Changes to the voice are prevalent and occur early in Parkinson’s disease. Correlates of these voice changes on four-dimensional laryngeal computed-tomography imaging, such as the inter-arytenoid distance, are promising biomarkers of the disease’s presence and severity. However, manual measurement of the inter-arytenoid distance is a laborious process, limiting its feasibility in large-scale research and clinical settings. Automated methods of measurement provide a solution. Here, we present a machine-learning module which determines the inter-arytenoid distance in an automated manner. We obtained automated inter-arytenoid distance readings on imaging from participants with Parkinson’s disease as well as healthy controls, and then validated these against manually derived estimates. On a modified Bland-Altman analysis, we found a mean bias of 1.52 mm (95% limits of agreement -1.7 to 4.7 mm) between the automated and manual techniques, which improves to a mean bias of 0.52 mm (95% limits of agreement -1.9 to 2.9 mm) when variability due to differences in slice selection between the automated and manual methods are removed. Our results demonstrate that estimates of the inter-arytenoid distance with our automated machine-learning module are accurate, and represents a promising tool to be utilized in future work studying the laryngeal changes in Parkinson’s disease.

## Introduction

Parkinson’s disease (PD) is a progressive neurodegenerative condition which leads to increasing disability from motor dysfunction as well as a variety of non-motor symptoms. The diagnosis of PD as well as assessment of its progression both remain primarily dependent on clinical assessment of motor function [1]. However, by the time patients with Parkinson’s disease (pwPD) can be diagnosed clinically, there is already a 50% loss of nigrostriatal dopaminergic neurons [2, 3]. Developing biomarkers which could diagnose PD earlier would make it possible to initiate disease-modifying therapies prior to the onset of advanced neurodegeneration. Furthermore, in trials of disease-modifying therapies, utilizing biomarkers which could track disease progression more accurately than current clinical assessment tools may allow for smaller sample sizes and shorter follow-up periods [4].

The voice changes seen in PD are a promising candidate for such a biomarker. Voice disorder is prevalent in the disease [5–7] and changes to the voice may herald the onset of the disease, manifesting several years prior to the limb dysfunction [8, 9]. While the voice changes of PD have been studied by numerous modalities including acoustic analysis and direct visualization [10], we utilized dynamic 4-dimensional computed-tomography (4D-CT) scans of the larynx to study the structural movement during phonation [11, 12]. 4D-CT is a CT technique which involves continuous image acquisition of anatomical structures over time (the ‘fourth dimension’), thereby creating a dynamic volume data set. This allows quantitative analysis of the motion of anatomical structures.

Parkinson’s disease is characterized by slowed movements, decrementing in amplitude and/or speed upon repetition (i.e. bradykinesia). We hypothesized that a repetitive vocalization task with laryngeal 4D-CT could reveal differences in arytenoid cartilage movements between pwPD and healthy control participants. In our prior work [11, 12], two key measurements were taken which were 1) the minimum distance between the arytenoid cartilages (denoted the inter-arytenoid distance or IAD), and 2) the glottic area. We previously demonstrated that changes to arytenoid cartilage posturing distinguish pwPD from healthy controls. pwPD have a reduction in the IAD and an increase in the glottic area during vocalization [11, 12]. We also found that as the condition advances over both time and clinical severity, the IAD progressively decreases while the glottic area increases [12].

The reduction in IAD suggests that the arytenoid cartilages are closer together in PD. This is counterintuitive in the presence of hypokinetic dysarthria. Reduced IAD in PD is consistent with prior laryngoscopic and EMG observations. In pwPD with asymmetric motor symptoms, laryngoscopic studies show that the arytenoid cartilage is hyperadducted on the side of the body which demonstrates more severe parkinsonism, causing its vocal fold to cross beneath the vocal fold on the contralateral side [13]. These observations are supported by laryngeal EMG studies of pwPD: the laryngeal adductors (thyroarytenoid, cricothyroid and lateral cricoarytenoid muscles) demonstrate increased spontaneous muscle activity at rest [14–17], while the sole abductor (posterior cricoarytenoid muscle) shows decreased activity [14]. The IAD only accounts for the sliding adduction movements of the arytenoid cartilages, but not their rotational movements. There may be reduction in arytenoid cartilage movement in other planes which our method of analysis is not able to measure. Measurement of the rotational movements would require landmarks such as the vocal and muscular processes to be marked, but these are not always reliably identified on CT. In our study, we were only able to reliably mark the base of the vocal processes [12]. As the vocal folds attach to the tip of the vocal processes, IAD as we measure it does not exactly correspond to vocal fold adduction.

Understanding the hypokinetic hypophonic dysarthria of PD is complicated by the laryngoscopic finding of glottic incompetence due to bowing of the vocal folds [13, 14, 18]. The increase in glottic area in pwPD despite a reduced IAD is a surrogate marker for vocal fold bowing [12] and this may explain the breathiness of the voice that can be appreciated in pwPD [19, 20].

4D laryngeal CT shows promise in both the diagnosis of PD, as well as in monitoring its progression. Co-existing vocal disorders or atypical parkinsonism may affect IAD and glottic area. We do not know if our findings are specific for PD as we excluded such patients in our study. Further controlled prospective research correlating IAD and glottic area with acoustic and perceptual voice analyses in parkinsonism (including atypical parkinsonian conditions) would be necessary before 4D laryngeal CT could be more widely adopted in a clinical or research setting.

Our study protocol required manually placing a fiducial marker on the most anteromedial aspect of each of the arytenoid cartilages, which would correspond with the base of the vocal process. The Euclidean distance between these markings was then derived to determine the IAD. On the other hand, the glottic area was automatically segmented using workstation software (IntelliSpace Portal, Philips Healthcare, Cleveland USA). Manual determination of the IAD at 100 ms intervals on the large volume 4D dynamic CT dataset during phonation is a laborious and time-consuming process, so automated means of measuring the IAD could facilitate the study of larger data sets. Placement of fiducial markers is also subjective and expected to have a degree of inter- and intra-observer variability–an issue which could be largely overcome through automated measurement techniques.

We previously described an automated method which used classical imaging processing techniques to detect the most anteromedial aspect of the arytenoid cartilages. Comparing the feature points identified by this automated rule-based approach to fiducial markers manually placed by investigators, an accuracy of 83.33% was achieved within an error tolerance of 15 pixels (equivalent to an anatomical length of 3.33 mm) [21]. This represents the error in the placement of the individual fiducial markers; the IAD between the fiducial markers was not calculated in this study. The mean IAD during a vocalization task in our cohort of pwPD of varying duration and severities was 4.24 mm, while it was 5.21 mm in control participants (comparing the unadjusted median)–a difference of only 0.97 mm [12]. In our study comparing pwPD with relatively early-stage disease against controls, adjusted for sex, the mean difference was 0.87 mm [11]. The error tolerance of 3.33 mm is therefore rather high given our findings of such small differences in the IAD between pwPD and healthy controls.

In this study, we employ a novel automated technique for deriving IAD estimates which differs in two key aspects from the method previously described. First, it employs a computer algorithm which is trained with machine-learning techniques to automatically identify the regions of interest, as compared to the fixed rule-based approach adopted previously. Next, it marks bounding boxes around the entirety of each arytenoid cartilage, in as many axial slices of the larynx as possible, for each imaging volume.

By processing a wider range of inputs, we expect that the machine-learning module will provide IAD estimates closer to those which are manually derived than the previous rule-based method. The machine-learning module considers factors both within and across image slices to accurately identify the position of the arytenoid cartilages. In contrast, the rule-based approach operates by only considering the individual axial slices around the plane of the glottis. This may lead to inaccuracies if there is misalignment of the imaging axis due to technical or anatomical reasons. Additionally, marking bounding boxes around the entirety of the arytenoid cartilages provides a measurement of their position. This offers the potential to conduct novel measurements of laryngeal function which have not been previously considered, such as measurement of movement in the vertical plane as can be seen in laryngeal tremor [22].

The aim of this study is to validate the IAD measurements derived from the automated detection of bounding boxes around the arytenoid cartilages by the machine learning module, against the current gold-standard of manual markings by investigators which we utilized in our prior work. In doing so, we intend to provide a basis for its use in future research using 4D dynamic laryngeal CT to study PD and other similar disorders.

## Materials and methods

### Patients and recruitment

We used the existing four-dimensional laryngeal CT image data set from our prior work. We recruited participants from the Movement Disorders clinic at Monash Medical Centre. Our initial study [11] recruited healthy controls as well as pwPD with early-stage disease, which we defined as a disease duration less than 6 years and a modified Hoehn and Yahr stage of 2.5 or lower. Our subsequent study [12] recruited pwPD with more advanced disease, which we defined as a disease duration of 5 years or greater and a Hoehn & Yahr stage of 3 or more. Control participants were not affected by Parkinson’s disease or other neurological disorders on clinical assessment by a neurologist. We excluded participants with respiratory or laryngeal disorders, as well as those with brain, head and neck cancers. From this data set, we randomly selected 4 pwPD with early-stage disease, 4 pwPD with more advanced disease and 4 healthy controls to validate the technique across the populations we have studied thus far.

### Protocol approvals, registrations and patient consents

All participants provided written informed consent in accordance with the Declaration of Helsinki. The Research Ethics Committee of Monash Health granted ethics approval (HREC approval number 11230B).

### Image acquisition and processing

Using a 320 multi-detector row CT (Aquilion One, Tokyo, Canon Medical Systems), non-contrast four-dimensional dynamic volume CT imaging of the larynx was acquired over an anatomical z-axis length of 16 cm without CT table movement. During a continuous imaging period of 5 seconds, participants were positioned supine and produced five quick and clear phonations of /i/ (‘eee’) at a comfortable speaking volume and pitch. Image acquisition was terminated before five seconds in those who had completed the vocalization task early. pwPD underwent imaging in a practically-defined ‘off’ state by withholding their usual PD medications overnight. The radiation dose is within the range of 0.8 to 2.0 mSv, depending on the amount of soft tissue in the neck [11]–a dose roughly equivalent to that of a standard CT head scan [23].

Continuous multiplanar images were produced at 100 ms intervals and were analyzed in the Neuroimaging Informatics Technology Initiative (NIfTI-1) image format at a resolution of 512 x 512 pixels and a voxel size of 0.222 x 0.222 x 0.5 mm^3^. Although our prior work [11, 12] only considered measurements taken during the vocalization phase, we performed manual and automated measurements of the IAD across the entire image acquisition period (including incidental periods of voice rest and respiration) in this validation to increase the generalizability of our results to different states of the arytenoid cartilages.

### Manual image analysis

**Fig 1:**
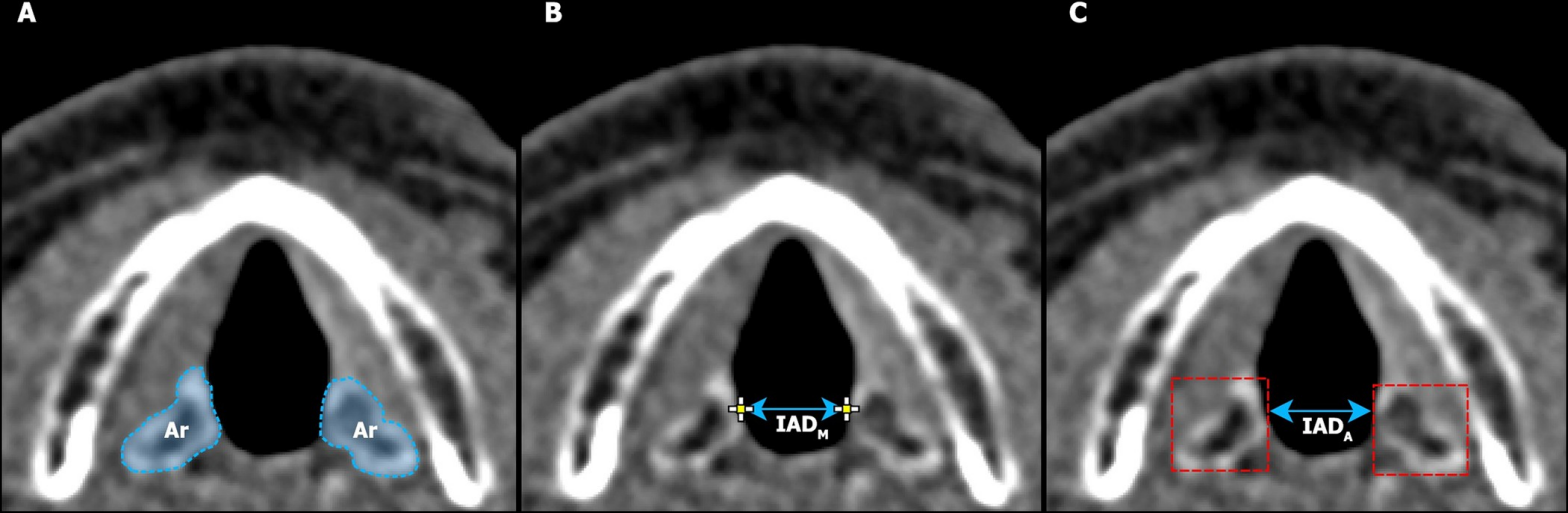
Illustrative diagrams comparing the image annotation techniques. 1A, axial slice through the larynx during voice rest, with the left and right arytenoid cartilages (Ar) marked in blue. 1B, fiducial markers manually placed at the anteromedial aspect of each arytenoid cartilage, with the corresponding IAD_M_ marked. 1C, bounding boxes marked in red around each arytenoid cartilage by the automated machine learning algorithm, with the corresponding IAD_A_ marked.

Images were manually annotated using the open-source ImageJ graphics software [24]. For each subject, at every timepoint within the vocalization period, the most medial aspect of each arytenoid cartilage was identified by scrolling through the image stack. A fiducial marker was then manually placed on the most medial aspect of each arytenoid cartilage. The manually estimated IAD (IAD_M_) was then calculated by finding the Euclidean distance between the two paired fiducial markers for every timepoint in each subject (see **Fig 1B**).

Rather than using the previously measured IAD data set, all images were re-annotated by investigator A.M for the purposes of this study to eliminate error due to inter-observer variability. Images were manually annotated in a randomized order across both subjects and timepoints to reduce auto-correlation. There was a risk of introducing bias by sequentially analyzing timepoints for a single subject, as the preceding annotations could influence the location of the markings for the following timepoint and thereby increasing dependence between data points.

### Automated image analysis

Images were analyzed using the automated image analysis technique developed by investigators N.D and colleagues [25]. The algorithm operated by detecting slices which contained the arytenoid cartilages and subsequently localized them before computing the IAD.

The image analysis algorithm consisted of a machine learning module which was designed using feedforward artificial neural nets that operated on orthogonal 2D views of the 4D CT data. The CT images acquired during the phonation task described previously, and those acquired during a standard breathing examination were combined to construct a dataset of 100 subjects. Only slices containing arytenoid cartilages were used for training the algorithm, with three-quarters of the included imaging data used in the training subset and one-quarter in the validation subset. An ubuntu 18.04 computing machine with Nvidia Quadro 6000 graphics card (24 gigabyte global memory) was used to train and validate the machine learning module. The implementation was done using the Pytorch library [26] and detectron2 [27] in Python.

Firstly, the algorithm marked bounding boxes around each arytenoid cartilage for each slice in which they were identified. Further, the arytenoid bounding boxes are localized in all the possible slices in the 3D volume for each timepoint. This process was repeated for each subject. The estimated IAD derived by the automated technique (IAD_A_) for a given timepoint was then calculated by finding the minimum distance between the paired structures in the x-axis/coronal plane (see **Fig 1C**).

The algorithm was also tuned to compute a ‘same slice’ automated IAD estimate (IAD_S_) on the same axial slice from which the IAD_M_ was derived. If the automated algorithm was unable to localize a pair of arytenoid cartilages on the slice marked by the manual observer, then that data point was excluded from analysis. Comparing the IAD_S_ to IAD_M_ removes the proportion of bias attributable to differences in slice selection between the manual and automated techniques. Therefore, the remaining bias is largely attributable to differences between the techniques in where the markings are placed within a slice.

### Statistical analysis

We performed all data processing, statistical analysis and creation of graphics with the R statistics and graphing software [28] and nlme package. The estimates obtained by the automated (IAD_A_) and same-slice automated (IAD_S_) techniques were compared to the estimates obtained by the manual technique (IAD_M_). We adjusted for repeated measures in our data by using linear mixed models to form regression lines. Similarly, considering the repeated measures, we utilized a modified Bland-Altman method to calculate the mean of the paired differences between the techniques, as well as their corresponding 95% limits of agreement.

The regression lines considered the IAD_M_ as explained by the fixed effect of either the IAD_A_ or IAD_S_, with random slopes and intercepts considered per participant to account for the repeated measures.

We performed a modified Bland-Altman analysis utilizing linear mixed models based on techniques previously described in the literature [29–31]. These modifications were necessary as our data consisted of repeated measures within individual subjects, thereby violating the assumption of independence inherent to the standard Bland-Altman method. Our models considered the paired differences between the IAD_M_ and IAD_A_, and IAD_M_ compared to IAD_S_, after considering inter-participant variation as a random effect. The R code for these analyses is provided in the **S1 Appendix**.

## Results

Imaging data was analyzed for 12 participants. 4 participants (1 female, 3 male) were healthy controls and had a mean age of 67 years (SD 7.4). 8 participants (2 female, 6 male) had a diagnosis of Parkinson’s disease and had a mean age of 71 years (SD 8.1). pwPD had a median disease duration of 94 months (IQR 51–126) and median UPDRS part-III score of 22 points (IQR 14–34).

Paired IAD_A_ and IAD_M_ measurements were taken on 560 CT volumes while only 515 paired measurements of the IAD_S_ and IAD_M_ were acquired. There were fewer IAD_S_ measurements as the automated technique did not detect the arytenoid cartilages on some slices marked by the manual observer.

**Fig 2:**
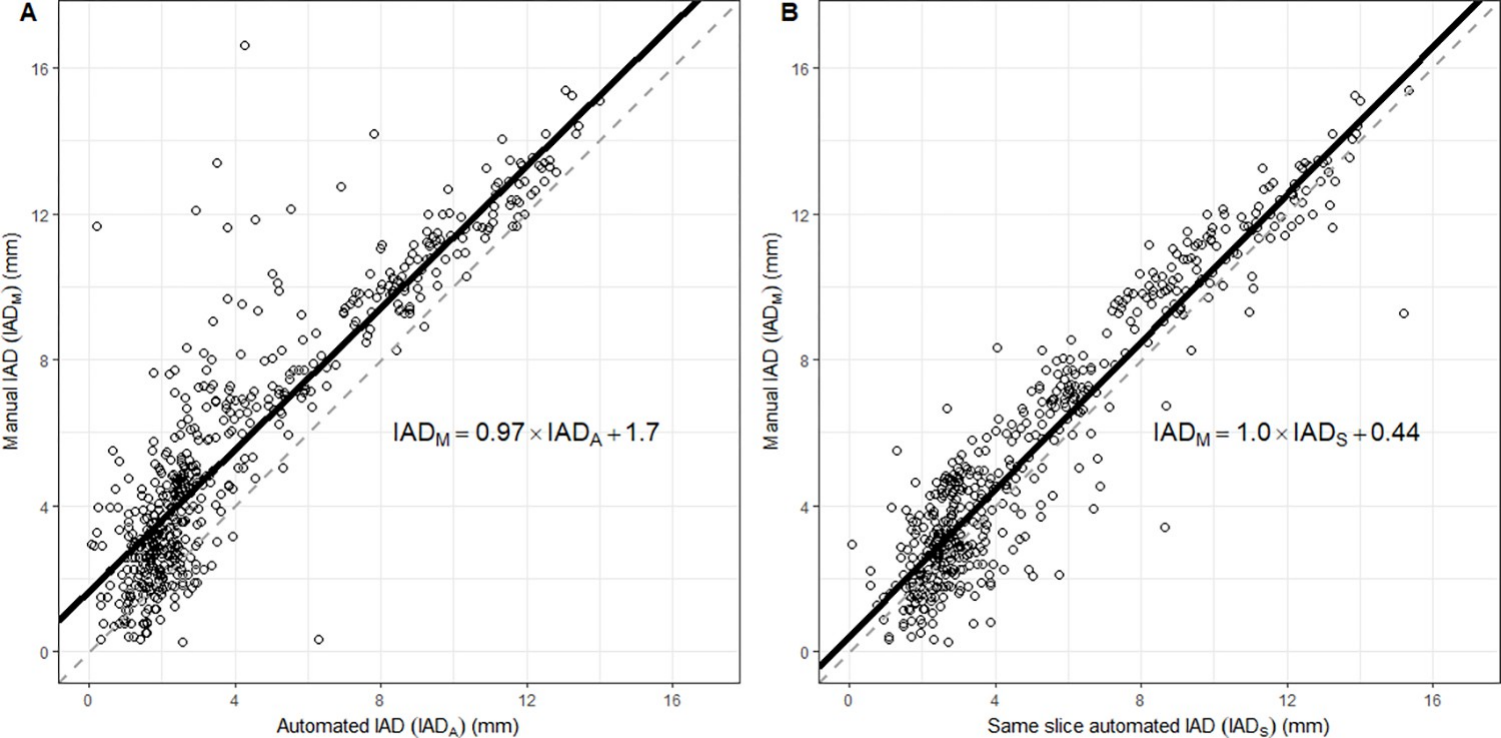
Scatter plots of the paired estimates. 2A compares the paired estimates of the IAD_M_ with IAD_A_, while 2B compares the paired estimates of IAD_M_ with IAD_S_. Regression lines are shown as solid black lines with their respective equations provided. The grey dashed lines represent the lines of equivalence.

**Fig 2** plots the IAD_M_ estimates paired against the IAD_A_ and IAD_S_. Regression lines considering repeated measures within subjects are overlayed.

**Fig 3:**
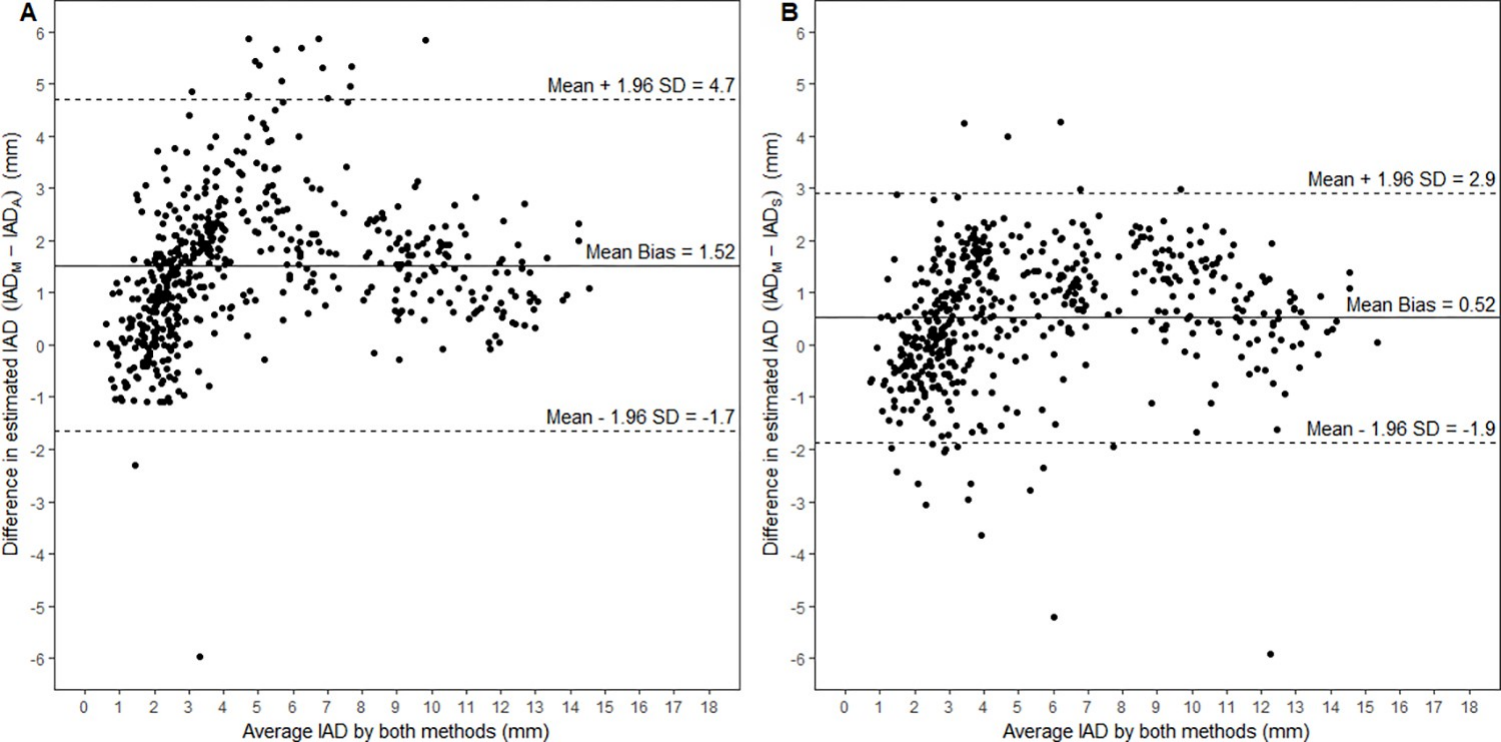
Bland-Altman plots. 3A compares the IAD_M_ against the IAD_A_, while 3B compares IAD_M_ to the IAD_S_. The mean bias (solid line), 95% upper and lower limits of agreement (dotted lines) are shown.

The modified Bland-Altman analysis accounting for repeated measures demonstrated that compared to the IAD_M_, the IAD_A_ had a mean difference of 1.52 mm with 95% limits of agreement from -1.7 to 4.7 mm. On the other hand, the IAD_S_ compared with the IAD_M_ showed a mean difference of 0.52 mm with 95% limits of agreement from -1.9 to 2.9 mm. The corresponding Bland-Altman plots are given in **Fig 3**. Further statistics pertaining to the Bland-Altman analysis are given in **Table 1**.

**Table 1:** Bland-Altman comparisons of the IAD_A_ and IAD_S_ against IAD_M_.

	IAD_A_	IAD_S_
Number of paired measurements	560	515
Mean bias (mm)	1.52	0.52
95% limit of agreements (mm)	-1.7 to 4.7	-1.9 to 2.9
Within participant standard deviation (mm)	1.45	0.94
Between participant standard deviation (mm)	0.73	0.77
Total standard deviation (mm)	1.6	1.2

## Discussion

This study evaluates the concordance of automated estimates of the IAD compared to those obtained through manual markings. We expect manual measurement of the IAD, as performed in our prior studies, to have a degree of inter- and intra-rater variability (although we have not previously formally determined this) and be subject to the influence of measurement error. This is a key motivation in exploring automated techniques of measurement, which would be more reproducible, less laborious and eliminate inter- and intra-rater variability. Our prior work utilized manual measurement of the IAD and by default is the current ‘gold standard’, but these measurements do not necessarily closely approximate the ground truth value.

In comparing the IAD_M_ with the IAD_A_, Bland-Altman analysis demonstrates a mean bias of 1.52 mm (i.e. the estimate of IAD_A_ is 1.52 mm less than the estimate of IAD_M_). Much of this discrepancy arises from differences introduced by differing slice selection between the automated and manual techniques. To remove this source of variability, we obtained the IAD_S_−automated measurements taken on the same CT slice as the IAD_M_. When comparing the IAD_M_ with the IAD_S_, there was an average discrepancy of 0.52 mm in the estimates given, which is less than 3 pixels difference. Thus, differences in slice selection by the manual and automated techniques accounts for a significant portion of the variability between the IAD_M_ and IAD_A_; an increase in the mean bias of 1.0 mm and a widening of the Bland-Altman limits of agreement by 1.6 mm.

The slice on which the IAD_M_ is measured is determined by gross visual inspection of where the arytenoid cartilages appear to be closest together. It is expected that the manual observer will not always select the slice where the ground truth IAD lies (i.e. where the pair of arytenoid cartilages are closest together). The automated technique calculates a distance between the arytenoid cartilages on every slice, with the minimum of this set of measurements taken as the IAD_A_. This approach would be expected to be more reliable in identifying the slice where the ground truth IAD lies than the manual technique. Having a manual observer perform measurements on every slice may achieve similar reductions in variability, but this would be unfeasible and markedly increase the work involved in what is already a laborious task. Ultimately, by removing variability due to imprecise slice selection by a manual observer, automated methods of IAD measurement may provide closer approximations of the ground-truth IAD.

The bounding box outputs of the machine-learning algorithm may also provide potential for more clinical applications. Capturing the positions of each arytenoid cartilage within three-dimensional space allows for novel measures to be considered, such as assessment of movements of the arytenoid cartilages in the vertical plane or z-axis over the imaging period. This could be applied to the study of vocal tremor, a phenomenon seen in PD which on laryngoscopic studies occurs predominantly in the vertical plane. Future work on laryngeal CT could assess these novel measures, which may offer additional benefits in the diagnosis or assessment of progression of PD beyond those which have been previously studied.

The 95% limits of agreement in comparing IAD_M_ to the IAD_A_ or IAD_S_ are still quite wide (-1.7 to 4.7 mm and -1.9 to 2.9 mm respectively). Random error is a major contributor to the variance in the agreement. A source of this random error is the subjective nature of fiducial placement which depends on where the observer judges the edge of the arytenoid cartilages to lie. On the other hand, the automated algorithm is consistent in its thresholds for edge detection. Whilst this normally distributed random error will be averaged out in a study such as ours using repeated measures, the automated algorithm will be preferred because of its consistency.

Limitations of fully automated techniques of measurement include their susceptibility to artifactual measurements. Inspection of **Fig 2A** shows numerous outliers in the upper left quadrant of the scatter plot, with the IAD_A_ estimate being substantially lower than the IAD_M_. Differences this large suggest that the estimates taken by the automated technique are artifactual, as it is unlikely the manual observer would have made such a substantial deviation from the ground truth. The automated algorithm selects the minimum of the IAD estimates across all measured slices for a given timepoint. Therefore, a single outlier which underestimates the ground truth IAD will be chosen, rendering the automated technique susceptible to artifacts of this nature. However, these outliers all occurred at high IAD_M_ readings, which correspond to periods of vocal rest where the vocal folds lie abducted. Therefore, this shortcoming of the automated algorithm is not relevant to studies (such as ours) [12] of vocal fold motion during phonation.

Further analysis to assess the performance of the automated method in specific populations would have been ideal, such as comparisons between sexes, or between participants with earlier and more advanced disease. However, this study was limited to only 12 participants, preventing meaningful sub-group analyses. Although the algorithm performs quite consistently across a wide range of IADs (aside from very high values as discussed above), it is possible that other factors such as anatomical differences between groups could lead to reduced performance. Performing a further validation study with greater patient numbers could permit such sub-group analyses.

We performed a modified Bland-Altman analysis utilizing linear mixed models given the repeated measures nature of our data. Regarding the construction of our linear mixed models, we considered participants as a random effect as suggested by Parker and colleagues [30] given the nature of our study design. As timepoints were only 100 ms apart, there would be a high degree of auto-correlation of the IAD over successive measures. Additionally, our participants included both healthy controls, as well as people with Parkinson’s disease covering a range of disease durations. Parkinson’s disease could affect phonation, but our sample was not large enough to consider it as a fixed effect. Therefore, we accounted for these differences by considering participant as a random effect.

Ultimately, the question remains whether automated methods of estimating the IAD are suitable to replace manual techniques in future research, and as a clinical biomarker. Based on our previous work, we found a ‘threshold of detection’ of early PD in a controlled study was a 0.87 mm sex-adjusted mean difference in IAD when compared to controls [11]. In this validation study, we found close agreement between a novel automated measurement technique and the manual approach used in that study. The IAD_A_ was consistently 1.52 mm less than the IAD_M_ across the entire range of measurements. Therefore, provided a single technique is applied in such repeated measures group studies, PD may be reliably detected. The automated method should also have greater consistency in edge detection and slice selection than manual fiducial placement. Thus, the IAD_A_ may more closely represent the ground truth IAD than IAD_M._ Automation would also facilitate analysis of much larger data sets, through which measures such as the IAD could be further validated, thereby fostering their broader adoption into research and clinical settings. Automation could also facilitate novel measures such as vertical motion of the arytenoid cartilages. After consideration of the above, we propose that automated techniques for estimating the IAD are a valid and superior tool for use in future work assessing the position and movement of the arytenoid cartilages during 4D laryngeal CT phonation imaging.

## Supporting information

S1 Appendix Modified Bland-Altman analysis using linear mixed models in R. Provided is the code used in the R software to perform the modified Bland-Altman analysis, which accounts for repeated measures within our data through the use of linear mixed models.
(PDF)

## References

[1] Postuma RB, Berg D, Stern M, Poewe W, Olanow CW, Oertel W, et al. MDS clinical diagnostic criteria for Parkinson’s disease. Mov Disord. 2015;30(12):1591–601. doi: 10.1002/mds.26424 . 26474316

[2] Kordower JH, Olanow CW, Dodiya HB, Chu Y, Beach TG, Adler CH, et al. Disease duration and the integrity of the nigrostriatal system in Parkinson’s disease. Brain. 2013;136(8):2419–31. doi: 10.1093/brain/awt192 . 23884810 PMC3722357

[3] Fearnley JM, Lees AJ. Ageing and Parkinson’s Disease: Substantia Nigra Regional Selectivity. Brain. 1991;114(5):2283–301. doi: 10.1093/brain/114.5.2283 . 1933245

[4] Terwee CB, Roorda LD, Knol DL, De Boer MR, De Vet HC. Linking measurement error to minimal important change of patient-reported outcomes. Journal of clinical epidemiology. 2009;62(10):1062–7. doi: 10.1016/j.jclinepi.2008.10.011 . 19230609

[5] Logemann JA, Fisher HB, Boshes B, Blonsky ER. Frequency and cooccurrence of vocal tract dysfunctions in the speech of a large sample of Parkinson patients. J Speech Hear Disord. 1978;43(1):47–57. doi: 10.1044/jshd.4301.47 . 633872

[6] Hartelius L, Svensson P. Speech and swallowing symptoms associated with Parkinson’s disease and multiple sclerosis: a survey. Folia Phoniatr Logop. 1994;46(1):9–17. doi: 10.1159/000266286 . 8162135

[7] Ho AK, Iansek R, Marigliani C, Bradshaw JL, Gates S. Speech impairment in a large sample of patients with Parkinson’s disease. Behav Neurol. 1999;11(3):131–7. . 22387592

[8] Harel B, Cannizzaro M, Snyder PJ. Variability in fundamental frequency during speech in prodromal and incipient Parkinson’s disease: a longitudinal case study. Brain Cogn. 2004;56(1):24–9. doi: 10.1016/j.bandc.2004.05.002 . 15380872

[9] Fereshtehnejad S-M, Yao C, Pelletier A, Montplaisir JY, Gagnon J-F, Postuma RB. Evolution of prodromal Parkinson’s disease and dementia with Lewy bodies: a prospective study. Brain. 2019;142(7):2051–67. doi: 10.1093/brain/awz111 . 31111143

[10] Ma A, Lau KK, Thyagarajan D. Voice changes in Parkinson’s disease: What are they telling us? J Clin Neurosci. 2020;72:1–7. doi: 10.1016/j.jocn.2019.12.029 31952969

[11] Perju-Dumbrava L, Lau K, Phyland D, Papanikolaou V, Finlay P, Beare R, et al. Arytenoid cartilage movements are hypokinetic in Parkinson’s disease: A quantitative dynamic computerised tomographic study. PLOS ONE. 2017;12(11):e0186611. doi: 10.1371/journal.pone.0186611 . 29099841 PMC5669420

[12] Ma A, Lau KK, Thyagarajan D. Radiological correlates of vocal fold bowing as markers of Parkinson’s disease progression: A cross-sectional study utilizing dynamic laryngeal CT. PLOS ONE. 2021;16(10):e0258786. doi: 10.1371/journal.pone.0258786 . 34653231 PMC8519464

[13] Hanson DG, Gerratt BR, Ward PH. Cinegraphic observations of laryngeal function in Parkinson’s disease. Laryngoscope. 1984; 94(3):348–353. doi: 10.1288/00005537-198403000-00011 6700351

[14] Gallena S, Smith PJ, Zeffiro T, Ludlow CL. Effects of levodopa on laryngeal muscle activity for voice onset and offset in Parkinson disease. J Speech Lang Hear Res. 2001; 44(6):1284–1299. doi: 10.1044/1092-4388(2001/100) 11776365

[15] Zarzur AP, Duprat Ade C, Cataldo BO, Ciampi D, Fonoff E. Laryngeal electromyography as a diagnostic tool for Parkinson’s disease. Laryngoscope. 2014; 124(3):725–729. doi: 10.1002/lary.24379 24105813

[16] Zarzur AP, Duprat AC, Shinzato G, Eckley CA. Laryngeal electromyography in adults with Parkinson’s disease and voice complaints. Laryngoscope. 2007; 117(5):831–834. doi: 10.1097/MLG.0b013e3180333145 17473678

[17] Ishii K, Kumada M, Ueki A, Yamamoto M, Hirose H. Involuntary expiratory phonation as a dose-related consequence of L-dopa therapy in a patient with Parkinson’s disease. Ann Otol Rhinol Laryngol. 2003; 112(12):1040–1042. doi: 10.1177/000348940311201208 14703107

[18] Blumin JH, Pcolinsky DE, Atkins JP. Laryngeal findings in advanced Parkinson’s disease. Ann Otol Rhinol Laryngol. 2004 Apr; 113(4):253–258. doi: 10.1177/000348940411300401 15112966

[19] Midi I, Dogan M, Koseoglu M, Can G, Sehitoglu MA, Gunal DI. Voice abnormalities and their relation with motor dysfunction in Parkinson’s disease. Acta Neurol Scand. 2008; 117(1):26–34. doi: 10.1111/j.1600-0404.2007.00965.x 18031561

[20] Wendler J. Stroboscopy. J Voice. 1992; 6(2):149–154.

[21] Desai N, Rao AS, Palaniswami P, Thyagarajan D, Palaniswami M. Arytenoid cartilage feature point detection using laryngeal 3D CT images in Parkinson’s disease. Annu Int Conf IEEE Eng Med Biol Soc; 2017 Jul; 1820–1823. doi: 10.1109/EMBC.2017.8037199 . 29060243

[22] Perez KS, Ramig LO, Smith ME, Dromey C. The Parkinson larynx: tremor and videostroboscopic findings. J Voice. 1996;10(4):354–61. doi: 10.1016/s0892-1997(96)80027-0 . 8943139

[23] Power SP, Moloney F, Twomey M, James K, O’Connor OJ, Maher MM. Computed tomography and patient risk: Facts, perceptions and uncertainties. World J Radiol. 2016; 8(12): 902–15. doi: 10.4329/wjr.v8.i12.902 . 28070242 PMC5183924

[24] Schneider CA, Rasband WS, Eliceiri KW. NIH Image to ImageJ: 25 years of image analysis. Nature methods. 2012;9(7):671–5. doi: 10.1038/nmeth.2089 . 22930834 PMC5554542

[25] Desai N. Automating Computed Tomography Analysis for Early Diagnosis of Neurological Diseases [dissertation]. Melbourne: University of Melbourne; 2020. Available from: http://hdl.handle.net/11343/241680.

[26] Paszke A, Gross S, Massa F, Lerer A, Bradbury J, Chanan G, et al. PyTorch: An Imperative Style, High-Performance Deep Learning Library. 2019 December 01. Available from: https://ui.adsabs.harvard.edu/abs/2019arXiv191201703P.

[27] Wu Y, Kirillov A, Massa F, Lo W-Y, Girshick R. Detectron2. 2019. Available from: https://github.com/facebookresearch/detectron2.

[28] R Core Team. R: A Language and Environment for Statistical Computing. Vienna, Austria: R Foundation for Statistical Computing; 2019.

[29] Myles PS, Cui J. I. Using the Bland–Altman method to measure agreement with repeated measures. BJA: British Journal of Anaesthesia. 2007;99(3):309–11. doi: 10.1093/bja/aem214 . 17702826

[30] Parker RA, Weir CJ, Rubio N, Rabinovich R, Pinnock H, Hanley J, et al. Application of Mixed Effects Limits of Agreement in the Presence of Multiple Sources of Variability: Exemplar from the Comparison of Several Devices to Measure Respiratory Rate in COPD Patients. PLOS ONE. 2016;11(12):e0168321. doi: 10.1371/journal.pone.0168321 . 27973556 PMC5156413

[31] Biancofiore G, Critchley LA, Lee A, Yang XX, Bindi LM, Esposito M, et al. Evaluation of a new software version of the FloTrac/Vigileo (version 3.02) and a comparison with previous data in cirrhotic patients undergoing liver transplant surgery. Anesthesia and analgesia. 2011;113(3):515–22. doi: 10.1213/ANE.0b013e31822401b2 . 21680855

