# Supplementary material for: Automated measurement of inter-arytenoid distance on 4D laryngeal CT: A validation study
Source: PLoS One. 2023 Jan 18;18(1):e0279927. doi: 10.1371/journal.pone.0279927 (PMC9847963; doi:10.1371/journal.pone.0279927)
Supplement: S1 Appendix — Provided is the code used in the R software to perform the modified Bland-Altman analysis, which accounts for repeated measures within our data through the use of linear mixed models. (PDF) [file pone.0279927.s001.pdf]

```
# Data structure: All data is stored in the data frame ‘Data’,
# in which the paired differences between the manual IAD and
# automated IAD are stored as ‘a.diff’, and those between the
# manual IAD and ‘same-slice’ automated IAD are stored as
# ‘s.diff’.


# Dependencies

library(nlme)


# Manual IAD vs automated IAD

a.lme<-lme(a.diff~1, random=~1|ID, data=Data,
na.action=na.omit)

a.withinsd<-as.numeric(VarCorr(a.lme)[2,2])

a.betweensd<-as.numeric(VarCorr(a.lme)[1,2])

a.totalsd<-sqrt((as.numeric(VarCorr(a.lme)[1,1])+as.numeric
(VarCorr(a.lme)[2,1])))

a.mean<-summary(a.lme)$coefficients$fixed[[1]]

a.lower<-a.mean-(1.96*a.totalsd)

a.upper<-a.mean+(1.96*a.totalsd)


# Manual IAD vs same-slice automated IAD

s.lme<-lme(s.diff~1, random=~1|ID, data=Data,
na.action=na.omit)

s.withinsd<-as.numeric(VarCorr(s.lme)[2,2])

s.betweensd<-as.numeric(VarCorr(s.lme)[1,2])

s.totalsd<-sqrt((as.numeric(VarCorr(s.lme)[1,1])+as.numeric
(VarCorr(s.lme)[2,1])))

s.mean<-summary(s.lme)$coefficients$fixed[[1]]

s.lower<-s.mean-(1.96*s.totalsd)

s.upper<-s.mean+(1.96*s.totalsd)
```
